# Biochar Organic Fertilizer Combined with Indigenous Microorganisms Enhances the Growth of Landscape Grass Cultivated in a Substrate Mixed with Iron Tailings and Mining Topsoil

**DOI:** 10.3390/plants13213042

**Published:** 2024-10-30

**Authors:** Xinyue Li, Xun Zhang, Jiaoyue Wang, Zhouli Liu, Hewei Song, Jing An

**Affiliations:** 1 Key Laboratory of Pollution Ecology and Environmental Engineering, Institute of Applied Ecology, Chinese Academy of Sciences, Shenyang 110016, China; lixinyue_2393@163.com (X.L.);; 2 College Environmental, Shenyang University, Shenyang 110044, China; 3 College of Life Science and Engineering, Shenyang University, Shenyang 110044, China; 4 Northeast Geological S&T Innovation Center of China Geological Survey, Shenyang 110000, China; 5 Key Laboratory of Black Soil Evolution and Ecological Effect, Ministry of Natural Resources, Shenyang 110000, China; 6 National-Local Joint Engineering Laboratory of Contaminated Soil Remediation by Bio-Physicochemical Synergistic Process, Shenyang 110142, China

**Keywords:** iron tails, waste utilization, landscape grass, rhizosphere improvement, ecological restoration

## Abstract

Iron tailings from the mining process occupy vast land areas and pose a significant ecological risk. In order to reuse iron tailings resources and carry out in situ ecological restoration of a mine, in this study, a medium of mixed iron tailings and mining topsoil (m:m = 3:1) was used to plant landscape grasses, including *Lolium perenne* L. *(L. perenne*), *Pennisetum alopecuroides* (L.) Spreng. *(P. alopecuroides*), *Melilotus officinalis* (L.) Lam. (*M. officinalis*), and *Medicago sativa* L. (*M. sativa*). Biochar and chicken manure were used as biochar organic fertilizers and indigenous microorganisms were isolated from the rhizosphere soil of tested grasses. They were applied to enhance landscape grass growth by regulating rhizosphere microbial communities and nutrient conditions. The results showed that the biochar organic fertilizers significantly promoted the growth of the four landscape grasses, notably *P. alopecuroides*, increasing plant height, root length, root weight, and leaf fresh weight by 169%, 60%, 211%, and 388%, respectively. Additionally, *L. perenne* exhibited the greatest height increase (10%) following the application of bacterial solutions. Moreover, indigenous bacterial solutions enhanced chlorophyll content and phenylalanine ammonia-lyase (PAL) activity, with *P. alopecuroides* showing the highest chlorophyll increase of 58% and *M. sativa* exhibiting a 30.58% rise in PAL activity. The biochar organic fertilizer also significantly elevated soluble protein content in *P. alopecuroides* and *M. sativa* by 195% and 152%, respectively. It also effectively enhanced peroxidase (POD) activity in Poaceae grasses by 120% to 160%. After adding indigenous microorganisms, the rhizosphere soil of the landscape grass showed the highest Shannon–Wiener diversity index, reaching 3.561. The rhizosphere soil of *M. officinalis* had the highest microbial richness, with a value of 39. Additionally, the addition of indigenous microorganisms increased the nitrogen, phosphorus, and potassium content of the four plants by 8–19%, 6–14%, and 8–18%, respectively. This study offers a new approach for managing mining waste and ecological restoration in mining areas.

## 1. Introduction

Iron ore mining is a critically important economic activity, serving as a foundation for modern industry and a primary sector in economic development globally [1]. However, significant waste is generated during mining, especially iron tailings. According to one report, the average iron ore grade is only 26%, producing approximately 1.5 tons of tailings per ton of refined iron [2]. Annually, the mining industry produces approximately 20 to 25 billion tons of mining waste globally, with iron tailings accounting for about 39% of all tailings [3,4]. As the leading solid waste produced after beneficiation, tailings accumulated in vast tailings ponds are susceptible to wind and water erosion [5], which are related to their characteristics of fine granularity, high alkalinity, low organic content, and poor water retention and permeability [6]. More importantly, the accumulation of iron tailings presents substantial challenges to the ecological environment within and surrounding the mining area, such as soil erosion, vegetation deterioration, and air pollution [7,8], which can even pose risks to human health [9]. Therefore, the recycling of iron tailings and the ecological restoration of tailing ponds are pressing issues in the field of environmental conservation.

The common recycling method for iron tailings resources is the production of construction materials, such as cement, road building materials, concrete fillers, and the production of porous ceramics [10]. Except for industrial fields, the application potential of iron tailings in agricultural and environmental fields must be addressed. For example, using iron tailings to prepare polymer materials can enhance soil environmental quality through improved water retention, stabilized soil structure, and adsorption of harmful substances [11]. Currently, as a soil amendment, iron tailings have been used in saline–alkali land improvement and landscape greening [12]. When iron tailings are used directly as a plant growth substrate, they often exhibit poor structure, low macronutrient content, extremely high pH, and high salinity [13]. Therefore, in practical applications, improving the properties of iron tailings through physical and chemical methods or biological amelioration techniques is usually necessary. Research has shown that applying soil amendments such as sand, vermiculite, sawdust, and organic compost can effectively enhance the physicochemical properties of iron tailings [14]. For example, Heiskanen J. et al. found that adding 5% compost to tailing soil could promote the growth of timothy grass and white clover [15]. Given the suitability of iron tailings for plant cultivation, using them as a substrate for in situ ecological restoration in mining areas shows considerable promise.

Ecological restoration is a cost-effective and environmentally friendly approach for restoring degraded land in metal mines, aiming to promote the recovery of natural ecosystems and mitigate land degradation caused by mining activities [16]. In ecological restoration, the selection and cultivation of vegetation play a crucial role. The introduction of vegetation can effectively mitigate the leaching and dispersion of contaminants, reduce soil and water pollution, and enhance soil structure through root stabilization [17]. Currently, arbor and shrubs have achieved some success in tailing restoration and have effectively improved the ecological environment of tailing areas [18,19]. However, these trees are less adaptable to the highly alkaline conditions of iron tailings, which need 50–70 cm of planted soil [20]. To reduce the costs of ecological restoration, it is necessary to continuously screen plants that are tolerant and suitable for the harsh environment of mining areas. Herbaceous plants, which are hardy and have lower substrate requirements, stabilize the soil with their roots and provide aesthetic value, making them a practical option for revegetation [21]. However, there is limited research on whether landscape grasses can be grown using iron tailings as a substrate and whether they can achieve ecological restoration of iron tailings.

In this study, in order to investigate the promoting effects of biochar organic fertilizer and microbial fluids on the growth of landscape plants in a cultivation substrate mixed with iron tailing sand and mine topsoil, native pioneer landscape plants (*Lolium perenne* L. (*L. perenne*), *Pennisetum alopecuroides* (L.) Spreng. (*P. alopecuroides*), *Melilotus officinalis* (L.) Lam. (*M. officinalis*), and *Medicago sativa* L. (*M. sativa*)) were selected, and an indigenous microbial solution was extracted from the rhizosphere soil of the test plants. Plant growth is not only evaluated by appearance indices, such as plant height, root length, fresh leaf weight, and root weight, but also by physiological parameters, including the content of chlorophyll, soluble protein, and antioxidant enzyme activity of the plants. Moreover, the rhizosphere soil nutrient content and microbial diversity in the cultivation substrate were researched. The results of this study can provide a scientific basis and technical support for in situ restoration of mining areas with iron tailings and native plants.

## 2. Results

### 2.1. The Impact of Biochar Organic Fertilizer Combined with Indigenous Microorganisms on Plant Growth Indices

**Figure 1:**
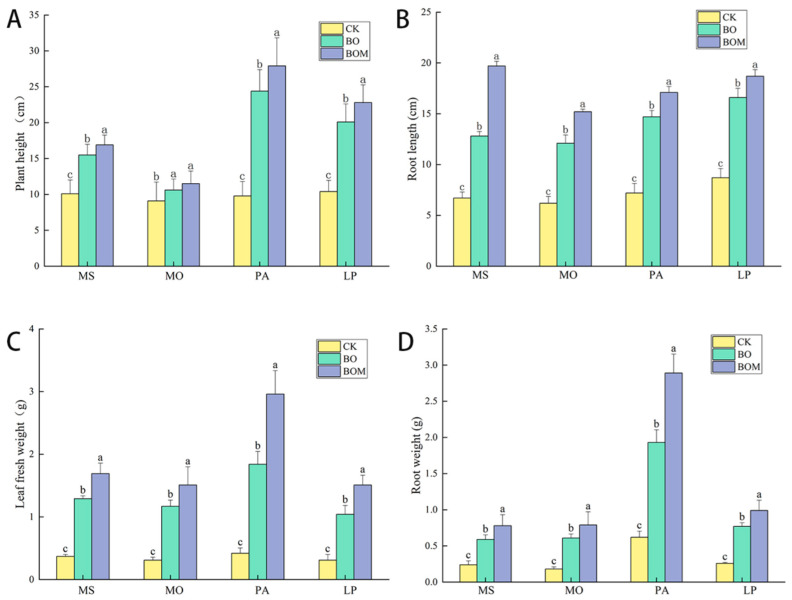
Plant growth indices of different treatment groups. ((**A**) plant height, (**B**) root length, (**C**) fresh leaf weight, (**D**) root weight). The values are expressed as the means (±standard error) from the experimental data (n = 3); different letters within the same column denote significant differences between treatments (*p* < 0.05). Notes: *Lolium perenne* L. (LP), *Pennisetum alopecuroides* (L.) Spreng. (PA), *Melilotus officinalis* (L.) Lam. (MO), and *Medicago sativa* L. (MS). Control check (CK), biochar organic fertilizers (BO), biochar organic fertilizers and indigenous microorganisms (BOM).

The addition of biochar organic fertilizer (BO) and indigenous microorganisms on top of BO (BOM) both improved the growth of the four experimental landscape grasses. The growth of the four test landscape grasses was improved by the treatments of BO and BOM (Figure S1). The plant growth indices of the landscape grasses under the different treatments are shown in Figure 1. Compared to the control check (CK), the plant height for *M. sativa*, *M. officinalis*, *P. alopecuroides*, and *L. perenne* increased significantly under the BO treatment by 5.4 cm, 1.5 cm, 16.6 cm, and 4.3 cm, respectively. Similarly, the root length, fresh leaf weight, and root weight of the four landscape grasses also increased significantly with the BO treatment. The fresh leaf weight of the grasses increased by more than 200%, while the root weight of the grasses increased by 146% to 211% in the BO group. The growth of gramineous plants was more responsive to BO, and the root length increased by 47% to 90% with BO addition, especially *P. alopecuroides.* Moreover, the screened indigenous microorganisms enhanced the growth-promoting effects of BO on the grasses. The plant height of the four species increased by 9%, 9%, 6%, and 10%, respectively, with *M. sativa* showing the largest change among the legumes and *P. alopecuroides* among the Poaceae grasses. The root length of the four grasses increased by 25%, 28%, 16%, and 23%, while their root weight increased by 18%, 22%, 25%, and 27%, respectively, with the most significant change observed in *L. perenne*.

### 2.2. The Effects of Biochar Organic Fertilizer Combined with Indigenous Microorganisms on Plant Physiological Parameters

#### 2.2.1. Chlorophyll Content

**Figure 2:**
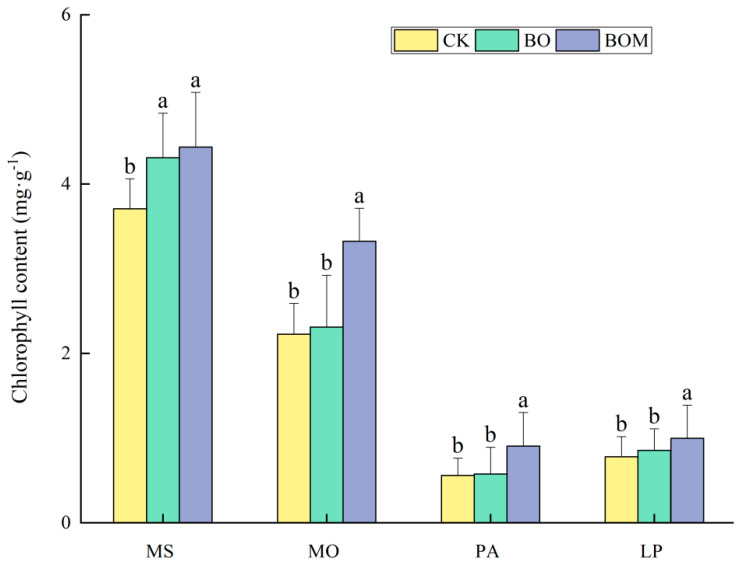
Chlorophyll content of landscape grasses in different treatments. The values are expressed as the means (±standard error) from the experimental data (n = 3); different letters within the same column denote significant differences between treatments (*p* < 0.05). Notes: *Lolium perenne* L. (LP), *Pennisetum alopecuroides* (L.) Spreng. (PA), *Melilotus officinalis* (L.) Lam. (MO), and *Medicago sativa* L. (MS). Control check (CK), biochar organic fertilizers (BO), biochar organic fertilizers and indigenous microorganisms (BOM).

The content of chlorophyll in grass leaves (*p* < 0.05) increased significantly with the addition of BO and BOM, as shown in Figure 2. Among the four test grasses, the increase in chlorophyll content in the leaves of *M. sativa* was most obvious when treated with BO compared to CK, up to 16%. For the other three grasses, however, the indigenous microorganisms contributed more to the increase in chlorophyll content in the leaves than BO. Comparing the BO and BOM treatment results, the addition of microbial solution significantly promoted the chlorophyll content in forage grass leaves. The chlorophyll content in *M. sativa*, *M. officinalis*, *P. alopecuroides*, and *L. perenne* leaves increased by 0.1 mg·g^−1^, 0.1 mg·g^−1^, 0.3 mg·g^−1^ and 0.1 mg·g^−1^, respectively. *P. alopecuroides* from the Poaceae family exhibited the most significant change, with a 58% increase in chlorophyll content after the addition of microbial solution.

#### 2.2.2. Soluble Protein Content

**Figure 3:**
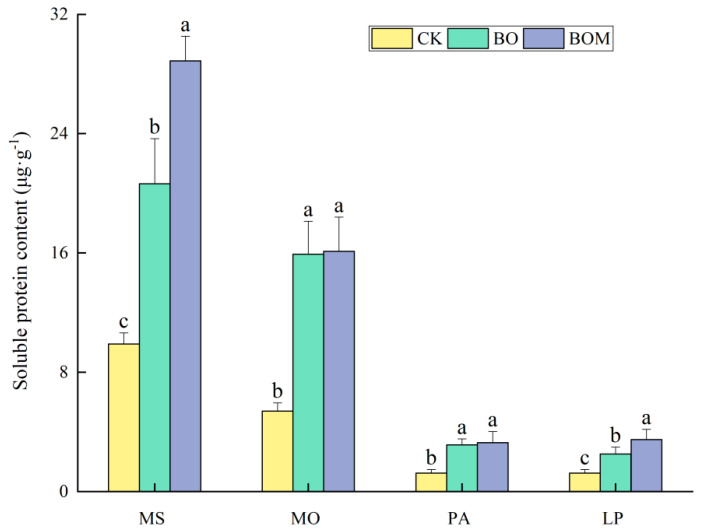
Soluble protein content in leaves of landscape grasses in different treatments. The values are expressed as the means (±standard error) from the experimental data (n = 3); different letters within the same column denote significant differences between treatments (*p* < 0.05). Notes: *Lolium perenne* L. (LP), *Pennisetum alopecuroides* (L.) Spreng. (PA), *Melilotus officinalis* (L.) Lam. (MO), and *Medicago sativa* L. (MS). Control check (CK), biochar organic fertilizers (BO), biochar organic fertilizers and indigenous microorganisms (BOM).

The soluble protein content in tested plants among different treatments is shown in Figure 3. It was found that the soluble protein content in the four landscape grasses was increased significantly by BO and BOM (*p* < 0.05). Specifically, the soluble protein content exhibited the highest increases in *L. perenne* and *M. sativa*, reaching growth rates of 195.11% and 151.53%, respectively. Comparing the BO, the addition of screened indigenous microorganisms solution resulted in increases of 8.2 mg·g^−1^, 0.2 mg·g^−1^, 0.1 mg·g^−1^, and 1 mg·g^−1^ in soluble protein content for *L. perenne*, *M. sativa*, *M. officinalis*, and *P. alopecuroides*, respectively. *L. perenne* and *M. sativa* showed the highest increases in soluble protein content, improving by 38% and 40% compared to BO.

#### 2.2.3. Antioxidant Enzyme Activity

**Figure 4:**
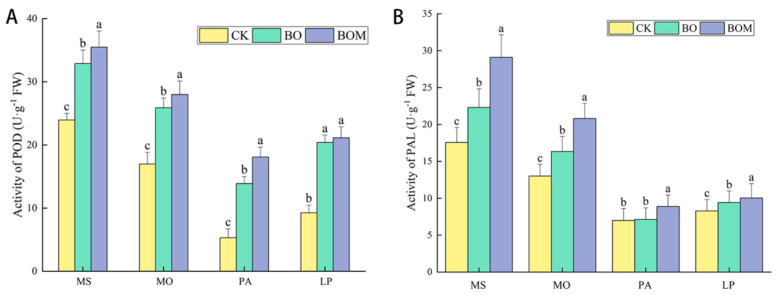
Antioxidant enzyme activity in the leaves of landscape grasses in different treatments. (**A**) Peroxidase (POD), (**B**) Phenylalanine ammonia-lyase (PAL). The values are expressed as the means (±standard error) from the experimental data (n = 3); different letters within the same column denote significant differences between treatments (*p* < 0.05). Notes: *Lolium perenne* L. (LP), *Pennisetum alopecuroides* (L.) Spreng. (PA), *Melilotus officinalis* (L.) Lam. (MO), and *Medicago sativa* L. (MS). Control check (CK), biochar organic fertilizers (BO), biochar organic fertilizers and indigenous microorganisms (BOM).

The typical antioxidant enzyme activities in the leaves of test grasses are shown in Figure 4. It was found that the peroxidase (POD) activity in the grasses was increased both by BO and BOM compared to CK, especially for gramineous plants. Compared to the control group, the activity of POD in *P. alopecuroides* and *L. perenne* increased by 161% and 120%, respectively, in the BO treatment group, while the POD activity in *M. sativa* and *M. officinalis* increased by 37% to 65%, respectively. Furthermore, adding indigenous microorganisms induced POD activity of the grasses in *P. alopecuroides*. The POD activity in *P. alopecuroides* was increased by 30% in the BOM group compared to the BO group. Similarly to the POD, the phenylalanine ammonia-lyase (PAL) activity in the test grasses was increased in the BO and BOM groups compared to CK, ranging from 2% to 27% (Figure 4B). Moreover, the BOM-treated *M. sativa*, *M. officinalis*, *P. alopecuroides*, and *L. perenne* exhibited increases in PAL activity of 31%, 28%, 24%, and 7%, respectively, compared to the BO group.

#### 2.2.4. Essential Nutrient Elements in the Landscape Grasses

**Figure 5:**
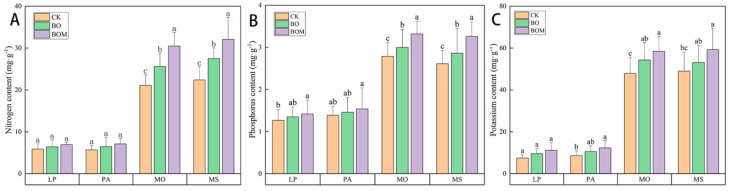
Nutrient element contents in pasture grasses of different treatment groups. (**A**) Nitrogen content, (**B**) Phosphorus content, (**C**) Potassium content. The values are expressed as the means (±standard error) from the experimental data (n = 3); different letters within the same column denote significant differences between treatments (*p* < 0.05). Notes: *Lolium perenne* L. (LP), *Pennisetum alopecuroides* (L.) Spreng. (PA), *Melilotus officinalis* (L.) Lam. (MO), and *Medicago sativa* L. (MS). Control check (CK), biochar organic fertilizers (BO), biochar organic fertilizers and indigenous microorganisms (BOM).

The nutrient element content in the tissues of test landscape grasses are shown in Figure 5. Compared to the CK group, nitrogen content in the leaf tissues of *P. alopecuroides*, *L. perenne*, *M. officinalis*, and *M. sativa* in the BO group increased by 9%, 14%, 21%, and 23%, respectively. The potassium content in the gramineous species *P. alopecuroides* and *L. perenne* exhibited significant increases of 29% and 23%, respectively, with BO treatment (Figure 5C). Moreover, the addition of indigenous bacteria further increased the content of nitrogen, phosphorus, and potassium in the test grasses. For example, compared to the BO group, the content of nitrogen, phosphorus, and potassium in *M. officinalis* increased 19%, 11%, and 8%, respectively, in the BOM group. Nitrogen and phosphorus content in leguminous grasses was more influenced by BOM treatment than in Poaceae species. There was a significant correlation between nitrogen content and the addition of BOM in leguminous grasses. The results showed that Poaceae grasses exhibited higher potassium uptake and utilization from the soil compared to leguminous plants.

### 2.3. The Impact of Microorganisms and Biochar Organic Fertilizer on the Rhizosphere Soil Microbial Community of Plants

The diversity indices, richness, and evenness of the four test grasses in the CK, BO, and BOM treatments were analyzed (shown in Table S1). It found that the microbial diversity and evenness in soil exhibited an upward trend following a 30-day cultivation period. However, the microbial diversity changed significantly with the species and growth conditions of plants.

#### 2.3.1. Diversity Index

**Figure 6:**
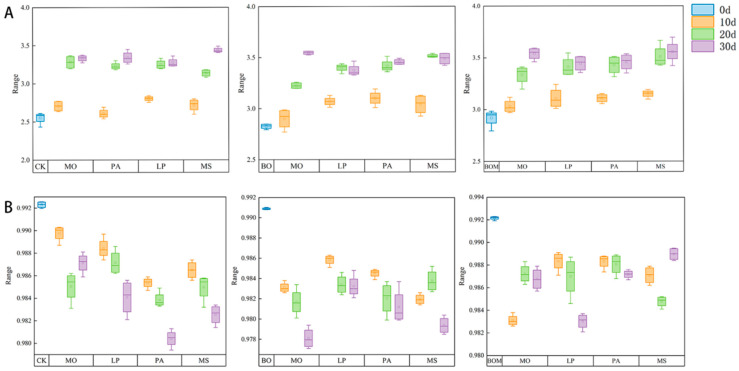
Diversity index for the rhizosphere soil microbes across different treatment groups. (**A**) Shannon–Wiener diversity index, (**B**) Simpson diversity index. The values are expressed as the means (±standard error) from the experimental data (n = 4). Notes: *Lolium perenne* L. (LP), *Pennisetum alopecuroides* (L.) Spreng. (PA), *Melilotus officinalis* (L.) Lam. (MO), and *Medicago sativa* L. (MS). Control check (CK), biochar organic fertilizers (BO), biochar organic fertilizers and indigenous microorganisms (BOM), d (day).

A separate analysis of each treatment group indicated that the Shannon–Wiener diversity index for the grass soil communities gradually increased over time (Figure 6A). From day 0 to day 30, the microbial diversity indices for the CK, BO, and BOM groups increased from 2.82 to 3.41, 2.95 to 3.54, and 2.99 to 3.56, respectively. When comparing data across different treatments, the soil microbial diversity in the experimental groups was higher overall than in the control group, with the BOM group exhibiting the highest values. This suggests a positive influence of the indigenous microbial solution and biochar organic fertilizer on the diversity of the soil microbial community.

Compared to the baseline at day 0, the Simpson diversity index generally showed a downward trend across all experimental groups (Figure 6B). This may be due to the Simpson index being sensitive to dominant species. Species richness increased as the growth period lengthened and microbial solutions, biochar, and organic fertilizers were introduced. However, the new species mainly consisted of rare or subdominant types, while the original dominant species’ numbers remained unchanged.

#### 2.3.2. Colony Similarity

**Figure 7:**
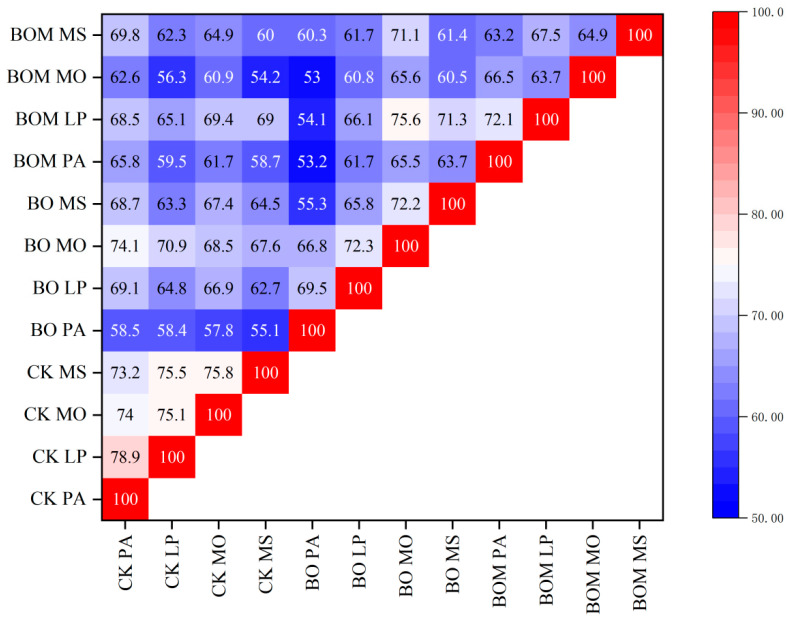
Heat map of soil colony similarity matrix across different treatment groups and plants at day 30. The values are expressed as the means (±standard error) from the experimental data (n = 3). Notes: *Lolium perenne* L. (LP), *Pennisetum alopecuroides* (L.) Spreng. (PA), *Melilotus officinalis* (L.) Lam. (MO), and *Medicago sativa* L. (MS). Control check (CK), biochar organic fertilizers (BO), biochar organic fertilizers and indigenous microorganisms (BOM).

Overall, the colony similarity among the four forage grasses in the CK group at day 30 was generally higher than in the experimental groups (Figure 7). Within the control group, the highest similarity was observed between *P. alopecuroides* and *L. perenne* at 79%, followed by *M. officinalis* and *M. sativa* with a similarity of 79%. In the BO group, the similarity between *M. officinalis* and *M. sativa* was 72%, while in the BOM group, the similarity between *P. alopecuroides* and *L. perenne* was 72%. This indicates that within the same treatment group, grasses from the same family exhibited higher microbial community similarities.

The addition of microbial solution, biochar, and organic fertilizer resulted in noticeable differences in soil colonies. The lowest similarity was observed in *P. alopecuroides* soil samples from the BO and BOM groups, at 53%. Comparing the similarity between the BO and BOM groups with the CK group, the introduction of exogenous substances significantly altered the composition of soil colonies.

## 3. Discussion

Landscape grasses can survive in mixed iron tailing substrates; however, their growth is often suboptimal due to the low nutrient content and poor aeration of the substrate [14]. The addition of biochar–organic fertilizer and soil microorganisms significantly affects the promotion of plant growth and development. Biochar possesses unique physical and chemical properties, such as low bulk density, porosity, high specific surface area, and alkalinity, which contribute to improving soil structure, enhancing aeration, and increasing water retention [22]. The application of chicken manure can further improve soil structure, enhancing the soil’s water, nutrients, air, and thermal conditions, making it looser and more fertile, thus benefiting the root growth of herbaceous plants [23]. Moreover, soil microorganisms decompose organic matter and interact with plant roots, promoting root development and enhancing the plant’s ability to absorb soil nutrients and water, leading to positive effects on plant growth indicators [24]. For instance, researchers found that adding plant growth-promoting bacteria under phosphorus-limited conditions in citrus soil enhanced leaf area, total volume, and root length [25]. This aligns with the findings of this study, where the combined use of indigenous bacterial solutions and biochar–organic fertilizer significantly improved the growth indicators of grasses planted in iron tailing substrates. Additionally, the root systems of herbaceous plants can penetrate the soil and stabilize soil particles, helping to maintain soil stability and reduce erosion, which is crucial for in situ restoration of tailing areas [26].

An increase in chlorophyll content not only enhances photosynthetic efficiency, allowing for more effective photosynthesis, but also improves a plant’s resilience to stress [27]. Consequently, effectively increasing chlorophyll content is essential for phytoremediation efforts in mining areas. Research by Siswanti D et al. revealed that the exogenous application of biofertilizers can substantially augment chlorophyll content in plants and mitigate the yellowing of fescue leaves [28]. This finding aligns with the observed increases in chlorophyll content indices in pasture in the current study, where the addition of indigenous bacterial solutions was particularly efficacious in enhancing the chlorophyll content in pasture leaves. Furthermore, the introduction of microorganisms and biochar organic fertilizer has been shown to elevate the soluble protein content and antioxidant enzyme activity in plant foliage, which positively influences plant resistance and physiological adaptability to complex soil environments [29]. This adaptability manifests in stronger growth, higher disease and pest resistance, and improved water-use efficiency, which further promotes increases in plant height and root length under optimal conditions [30]. The results of this study confirmed the beneficial effects of microorganisms and biochar–organic fertilizer on the soluble protein content and antioxidant enzyme activity in grass leaves. Specifically, microorganisms had a more pronounced effect on PAL activity, while biochar–organic fertilizer had a more significant impact on soluble protein content and POD activity.

Enhancing soil microbial diversity and richness is crucial for plant health and soil fertility [31]. Different microorganisms offer a variety of ecological services to plants, such as nutrient cycling and disease resistance [32]. A rich microbial community can stimulate plant growth, and higher soil microbial diversity increases the potential for complementary functions and metabolic pathways within the plant–microbe system [33]. Studies have shown that the positive effects of microbial diversity on plant growth intensify over time, with better plant growth leading to higher levels of growth-promoting bacteria, thus creating a beneficial feedback loop in the plant–microbe system [34]. Increased microbial richness in soil typically indicates a greater variety of microorganisms involved in nutrient cycling [35]. For example, one study found that treatment of rosette plants with bacterial solutions of varying richness resulted in increased rosette diameter as bacterial richness increased [36]. Furthermore, higher soil microbial richness may enhance plant disease resistance by inhibiting the formation of plant pathogens, thereby promoting healthy plant growth [37]. Improving microbial diversity and richness also contributes to greater resilience of soil ecosystems [38]. Biochar, with its large specific surface area, provides an excellent habitat for soil microorganisms, increasing their activity and abundance [39]. The results of this study indicate that the addition of indigenous bacterial solutions, biochar, and organic fertilizer significantly improved both the diversity and richness of rhizosphere soil microorganisms. Additionally, in the face of external pressures such as climate and soil changes, systems with higher microbial diversity and richness are better able to maintain relative stability in plant growth [40]. This enhancement aligns with the study’s goal of promoting revegetation in iron tailing areas.

Efficient soil nutrient absorption is crucial for plant growth. The structure of biochar aids in moisture retention and nutrient adsorption in the soil, thereby reducing nutrient loss [41]. Organic fertilizers contribute a substantial reservoir of microbial and essential nutrients [42]. The combined use of biochar and organic fertilizer can improve soil structure and enhance nitrogen retention and supply [43]. Additionally, the high phosphorus content in organic fertilizer contributes to increased phosphorus availability in the soil, while the elevated potassium levels help strengthen the soil’s capacity to retain and release this essential nutrient [44]. Improvements in soil conditions have a direct impact on nutrient absorption by plants [45]. In this study, the nutrient content in grasses treated with biochar–organic fertilizer was higher than in the control group. This may be attributed to the enhanced nutrient levels in the soil, which improved the grasses’ ability to absorb nutrients, thereby promoting growth. Furthermore, soil microorganisms can increase the availability of nitrogen, phosphorus, and potassium, enhancing the physiological activity of plants and fostering grass growth [46]. Leguminous plants, such as *M. officinalis* and *M. sativa*, used in this experiment can form symbiotic relationships with nitrogen-fixing rhizobia, resulting in root nodules that further increase soil nitrogen levels. This symbiotic relationship enhances soil nutrients and promotes plant growth, potentially due to the addition of indigenous bacterial solutions that boost the symbiosis with rhizobia or mycorrhizae [47], aligning with the observed increase in nitrogen content in grasses treated with indigenous bacterial solutions in this study. Furthermore, some microorganisms secrete organic acids and enzymes that dissolve phosphorus fixed in soil particles, converting organic phosphorus into inorganic forms that are more readily absorbed by plants [48]. Additionally, microorganisms participate in the decomposition of organic matter, releasing potassium that can be mineralized into a form available for plant uptake, thus increasing the soil’s exchangeable potassium content and supporting plant growth [49]. Researchers have noted that inoculating potassium-solubilizing bacteria improves potassium utilization in the soil [50], and plants treated with these bacteria exhibit higher photosynthetic rates and leaf area compared to controls [51]. Tests in this study revealed that the indigenous bacterial solutions contained strains capable of solubilizing phosphorus, fixing nitrogen, and dissolving potassium. The increased presence of these functional microorganisms further enhanced the availability of nitrogen, phosphorus, and potassium in the soil, promoting nutrient absorption by plants and consequently boosting plant growth and development. The test results of this study showed that the indigenous bacterial solutions contained strains capable of solubilizing phosphorus, fixing nitrogen, and dissolving potassium. The increased presence of these functional microorganisms further enhanced the availability of nitrogen, phosphorus, and potassium in the soil, promoting nutrient absorption by plants and consequently boosting plant growth and development.

## 4. Materials and Methods

### 4.1. Experimental Material

Iron tailings from the DaGushan mining area (41°2′39″ N, 123°4′16″ E) and mining topsoil from the dumpsite had pH values of 9.1 and 7.2, respectively. Total nitrogen content was 0.8 g·kg^−1^ and 2.3 g·kg^−1^. The forage grass seeds selected included the legumes *M. sativa* and *M. officinalis* and grasses *L. perenne* and *P. alopecuroides*, all purchased from Lantian Seed Industry. The biochar used was straw biochar prepared by the research team, with a pH of 9.51. Chicken manure was collected from the Sujia Tun chicken farm and naturally air-dried. It had a pH of 5.96, an organic matter content of about 63%, and a phosphorus content of about 2.41%.

### 4.2. Method of Compounding Culture Substrate

In the CK, the ratio of iron tailings to topsoil was 3:1. In the BO, the ratio of iron tailings to topsoil to biochar was 3:1:0.3, with 8 g of chicken manure added per pot. In the BOM, 120 mL of indigenous bacterial solution was applied every 10 days on a BO basis. All the culture substrates were mixed well and transferred to pots with 500 g per pot.

### 4.3. Method for the Preparation of Indigenous Microbial Formulations from Plant Rhizosphere Soil

The topsoil was passed through a 2 mm sieve, and 10 g was weighed and placed in a 150 mL conical flask. Then, 100 mL of sterile water was added, and the mixture was shaken thoroughly to create a soil stock solution, which was allowed to stand for 30 min. The supernatant was carefully aspirated into a clean centrifuge tube and centrifuged at 10,000 rpm for 1 min. After discarding the waste liquid, 1 mL of sterile water was added in a sterile environment, and the mixture was thoroughly mixed by pipetting. In an aseptic environment, 1 mL of the treated soil stock solution was added to a 150 mL Erlenmeyer flask containing sterilized beef extract–peptone liquid medium. This flask was placed in a shaker at 30 °C and 190 rpm for 48 to 72 h or until the medium became turbid. Once turbidity was achieved, 300 μL of the bacterial solution was added to another 150 mL of sterilized beef extract–peptone liquid medium and incubated for 12 to 24 h until reaching the logarithmic phase (OD_600_ approximately 1.0). The bacterial solution was then diluted 1:100, resulting in the indigenous bacterial solution used in subsequent experiments. High-throughput testing analyzed the microbial genera contained in the indigenous microbial solution (Figure S2).

### 4.4. Pot Experiment

In each pot, 50 viable seeds were evenly sown, and four different landscape grasses, *L. perenne*, *P. alopecuroides*, *M. officinalis*, and *M. sativa*, were planted individually. After seeding, a 0.5 cm layer of topsoil was spread over the surface, followed by adequate watering to ensure permeation. Each treatment was replicated three times. The potted plants were then placed in a constant-temperature growth chamber set at 27 °C, with a photoperiod of 12 h light and 12 h dark, and were watered regularly. Soil samples were collected every 10 days and stored in sealed bags at −80 °C for subsequent analysis. After 30 days, forage grass samples were collected, and both soil and plant samples were subjected to data measurement and analysis.

### 4.5. Methods for the Detection of Plant Growth and Development Indicators

Plant height was measured from the surface of the potting soil to the highest point of natural leaf extension. Root length was determined from the soil surface to the furthest point of natural root extension. Fresh leaf weight was calculated as the mass of the above-ground parts per plant, and root weight was measured as the mass of the belowground parts following soil removal.

Chlorophyll content was assessed via the 80% acetone extraction method [52]. Soluble protein content was determined using the Coomassie brilliant blue G-250 method [53]. POD activity was measured using the guaiacol method [54]; and PAL activity was determined using spectrophotometry [55].

Total nitrogen in plants was determined by the Kjeldahl digestion method [56], total phosphorus content via the molybdenum antimony colorimetric method [57], and total potassium content through flame photometry [58].

### 4.6. Detection Methods for Rhizosphere Soil Microbial Content

Soil samples were processed for total DNA extraction using an MP FastDNA Spin kit for Soil (MP Biomedicals, Santa Ana, CA, USA), following the manufacturer’s instructions. DNA extracted was then amplified for the bacterial V3 region by PCR, and the amplification products were subjected to denaturing gradient gel electrophoresis (DGGE). The gel bands were analyzed to assess microbial diversity.

The Shannon–Wiener diversity index (H′) and the Simpson diversity index (D) were used to reflect the level of species diversity in the ecosystem [59]. Richness (S) indicates the number of different species present within the microbiome, corresponding to the number of bands in the gel lanes. Evenness (E) describes the distribution of individual counts among species within the microbial community, reflecting the uniformity of individual numbers among species [60]. P_i_ is the number of individuals of the ith species as a proportion of the total number.

(1) $$H\prime =-\sum_{i=1}^{s}p_{i}\ln p_{i}$$

(2) $$E=\frac{H\prime }{\ln S}$$

(3) $$D=1-\sum_{i=1}^{s}{Pi}^{2}$$

### 4.7. Data Processing and Analysis

Data were analyzed using Excel and further processed and visualized using Origin 2021. Gel images were analyzed using Quantity One, and significance was assessed using SPSS 26. The data are presented as means ± standard deviation from three replicates. A one-way analysis of variance was used to evaluate the results, and differences between treatment means were determined using Tukey’s HSD test at a significance level of *p* < 0.05.

## 5. Conclusions

Using indigenous microbial solutions and biochar organic fertilizers significantly enhanced the growth of landscape grass using ferric tail sand as a substrate. The study’s findings indicate that the introduction of indigenous microorganisms and biochar organic fertilizers markedly increased root length, root mass, plant height, leaf fresh weight, chlorophyll content, soluble protein content, and antioxidant enzyme activities in forage grasses. Additionally, these amendments substantially affected the soil microbial community, augmenting the diversity index, richness, and evenness of soil microorganisms, and fostering the development of a robust soil microbial community. Crucially, these interventions were pivotal in enhancing the plants’ absorption and utilization of ammonium nitrogen, available phosphorus, and readily available potassium. Furthermore, the utilization of herbaceous plants has been proven to effectively reduce the costs associated with ecological restoration of mining sites and provides theoretical support for the improved realization of green restoration in mine-affected ecological environments.

## Data Availability

Data will be made available on request.

## Supplementary Materials

The following supporting information can be downloaded at: https://www.mdpi.com/article/10.3390/plants13213042/s1, Figure S1. The growth of the grasses under the treatments of CK, BO and BOM. (A: *Pennisetum alopecuroides* (L.) Spreng. B: *Lolium perenne* L. C: *Melilotus officinalis* (L.) Lam. D: *Medicago sativa* L.). Notes: Control check (CK), biochar organic fertilizers (BO), biochar organic fertilizers and indigenous microorganisms (BOM). Figure S2. Genera of bacteria contained in indigenous bacterial fluids. Table S1. The richness and evenness of rhizosphere soil microbial communities for landscape grasses under CK, BO and BOM treatment. Notes: *Lolium perenne* L. (LP), *Pennisetum alopecuroides* (L.) Spreng. (PA), *Melilotus officinalis* (L.) Lam. (MO), and *Medicago sativa* L. (MS). Control check (CK), biochar organic fertilizers (BO), biochar organic fertilizers and indigenous microorganisms (BOM). The values are expressed as the means (±standard error) from the experimental data (n = 3). Table S2. Plant growth indicators. Notes: *Lolium perenne* L. (LP), *Pennisetum alopecuroides* (L.) Spreng. (PA), *Melilotus officinalis* (L.) Lam. (MO), and *Medicago sativa* L. (MS). Control check (CK), biochar organic fertilizers (BO), biochar organic fertilizers and indigenous microorganisms (BOM). The values are expressed as the means (±standard error) from the experimental data (n = 3). Table S3. Plant physiological and biochemical indicators. Notes: *Lolium perenne* L. (LP), *Pennisetum alopecuroides* (L.) Spreng. (PA), *Melilotus officinalis* (L.) Lam. (MO), and *Medicago sativa* L. (MS). Control check (CK), biochar organic fertilizers (BO), biochar organic fertilizers and indigenous microorganisms (BOM). The values are expressed as the means (±standard error) from the experimental data (n = 3). Table S4. Nutrient content in plants. Notes: *Lolium perenne* L. (LP), *Pennisetum alopecuroides* (L.) Spreng. (PA), *Melilotus officinalis* (L.) Lam. (MO), and *Medicago sativa* L. (MS). Control check (CK), biochar organic fertilizers (BO), biochar organic fertilizers and indigenous microorganisms (BOM). The values are expressed as the means (±standard error) from the experimental data (n = 3). Table S5. Shannon-Wiener and Simpson Diversity Index. Notes: *Lolium perenne* L. (LP), *Pennisetum alopecuroides* (L.) Spreng. (PA), *Melilotus officinalis* (L.) Lam. (MO), and *Medicago sativa* L. (MS). Control check (CK), biochar organic fertilizers (BO), biochar organic fertilizers and indigenous microorganisms (BOM). The values are expressed as the means (±standard error) from the experimental data (n = 4).

## Abbreviations

CK	control check
BO	biochar organic fertilizer
BOM	biochar organic fertilizer and indigenous microorganisms
PAL	phenylalanine ammonia-lyase
POD	peroxidase
*L. perenne*, LP	*Lolium perenne* L.
*M. officinalis*, MO	*Melilotus officinalis* (L.) Lam.
*M. sativa*, MS	*Medicago sativa* L.
*P. alopecuroides*, PA	*Pennisetum alopecuroides* (L.) Spreng.
