# Supplementary material for: Biochar Organic Fertilizer Combined with Indigenous Microorganisms Enhances the Growth of Landscape Grass Cultivated in a Substrate Mixed with Iron Tailings and Mining Topsoil
Source: Plants (Basel). 2024 Oct 30;13(21):3042. doi: 10.3390/plants13213042 (PMC11548118; doi:10.3390/plants13213042)
Supplement: Supplementary file 1 [file plants-13-03042-s001.zip › plants-3256420-supplementary.pdf]

Biochar Organic Fertilizer Combined with Indigenous Microorganisms Enhances the Growth of Landscape Grass Cultivated in a Substrate Mixed with Iron Tailings and Mining Topsoil

Xinyue Li [1,2], Xun Zhang [2], Jiaoyue Wang [1], Zhouli Liu [3,4,5], Hewei Song [1] and Jing An [1,6,]

[1] Key Laboratory of Pollution Ecology and Environmental Engineering, Institute of Applied Ecology, Chinese Academy of Sciences, Shenyang 110016, China
[2] College Environmental of Shenyang University, Shenyang 110044, China
[3] College of Life Science and Engineering, Shenyang University, Shenyang 110044, China
[4] Northeast Geological S&T Innovation Center of China Geological Survey, Shenyang 110000, China
[5] Key Laboratory of Black Soil Evolution and Ecological Effect, Ministry of Natural Resources, Shenyang 110000, China
[6] National-Local Joint Engineering Laboratory of Contaminated Soil Remediation by Bio-Physicochemical Synergistic Process, Shenyang 110142, China

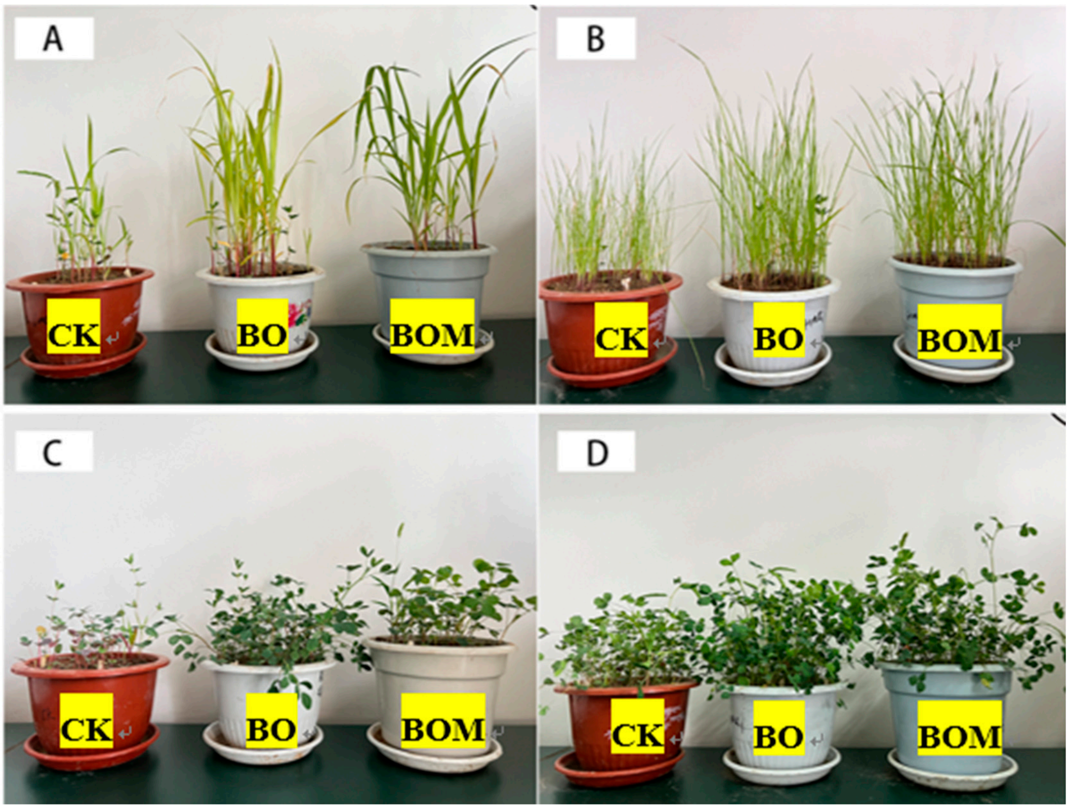

Figure S**1.** The growth of the grasses under the treatments of CK, BO and BOM. (A: *Pennisetum alopecuroides* (L.) Spreng. B: *Lolium perenne* L. C: *Melilotus officinalis* (L.) Lam. D: *Medicago sativa* L.). Notes: Control check (CK), biochar organic fertilizers (BO)， biochar organic fertilizers and indigenous microorganisms (BOM).

Table S1. The richness and evenness of rhizosphere soil microbial communities for landscape grasses under CK, BO and BOM treatment. Notes: *Lolium perenne* L. (LP)*, Pennisetum alopecuroides* (L.) Spreng. (PA)*, Melilotus officinalis* (L.) Lam. (MO)*, and Medicago sativa* L. (MS). Control check (CK), biochar organic fertilizers (BO), biochar organic fertilizers and indigenous microorganisms (BOM). The values are expressed as the means (± standard error) from the experimental data (n = 3).

Processing group name	Time（day）	Landscape Grass Namess	Richness (S)	Evenness (E)
CK	0		21	0.851±0.001
	10	PA	25	0.804±0.004
		LP	24	0.885±0.002
		MO	22	0.853±0.008
		MS	22	0.841±0.002
	20	PA	27	0.935±0.008
		LP	26	0.930±0.003
		MO	27	0.955±0.005
		MS	24	0.970±0.009
	30	PA	30	0.946±0.002
		LP	28	0.943±0.001
		MO	29	0.944±0.002
		MS	30	0.963±0.002
BO	0		22	0.912±0.003
	10	PA	25	0.953±0.007
		LP	24	0.973±0.003
		MO	22	0.929±0.002
		MS	24	0.920±0.002
	20	PA	29	0.994±0.001
		LP	30	0.973±0.002
		MO	28	0.960±0.006
		MS	26	0.900±0.001
	30	PA	31	0.993±0.001
		LP	33	0.991±0.001
		MO	35	0.986±0.002
		MS	34	0.973±0.005
BOM	0		21	0.961±0.002
	10	PA	26	0.987±0.002
		LP	24	0.996±0.003
		MO	23	0.970±0.005
		MS	25	0.991±0.001
	20	PA	31	0.972±0.004
		LP	34	0.995±0.002
		MO	32	0.984±0.002
		MS	30	0.964±0.002
	30	PA	34	0.983±0.002
		LP	34	0.996±0.003
		MO	39	0.988±0.002
		MS	37	0.992±0.001

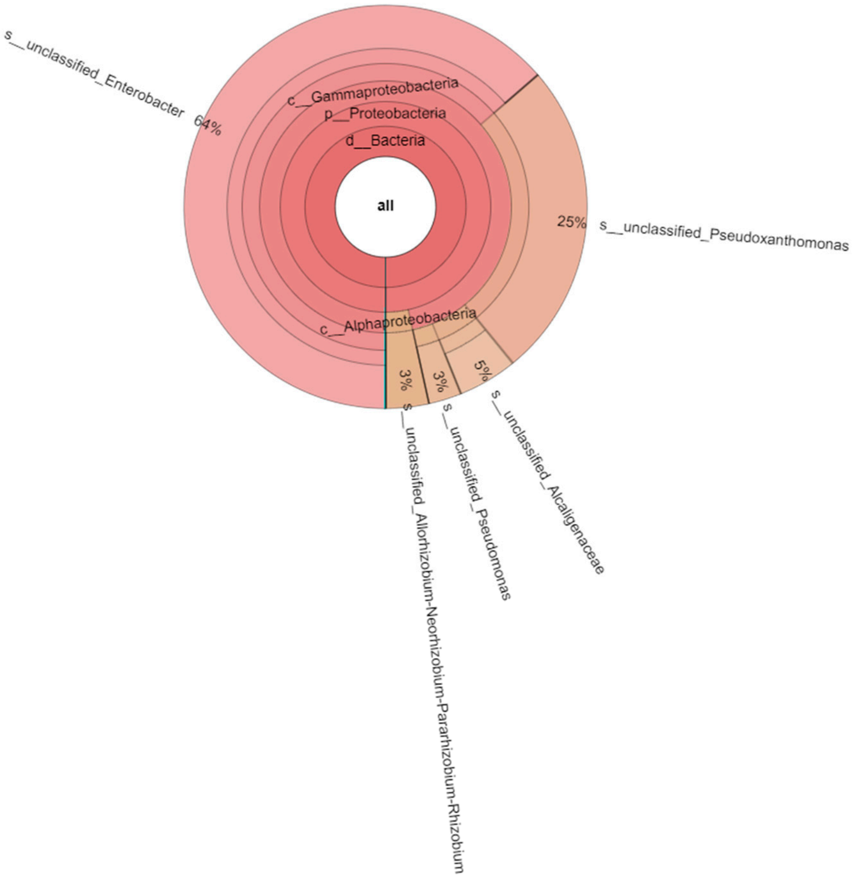

Figure S2. Genera of bacteria contained in indigenous bacterial fluids.

Table S2. Plant growth indicators. Notes: *Lolium perenne* L. (LP), *Pennisetum alopecuroides* (L.) Spreng. (PA), *Melilotus officinalis* (L.) Lam. (MO), *and Medicago sativa* L. (MS). Control check (CK), biochar organic fertilizers (BO), biochar organic fertilizers and indigenous microorganisms (BOM). The values are expressed as the means (± standard error) from the experimental data (n = 3).

Processing group name	Landscape Grass Names	Plant height (cm)	Root weight (g)	Leaf fresh weight (g)	Root length (cm)
CK	MS	10.13±1.39	0.24±0.06	0.37±0.03	6.7±0.59
	MO	9.13±1.44	0.18±0.03	0.32±0.05	6.2±0.65
	PA	9.77±1.52	0.62±0.08	0.42±0.08	0.42±0.08
	LP	10.4±1.556	0.26±0.11	0.31±0.09	8.6±0.92
BO	MS	15.53±1.52	0.59±0.07	1.29±0.04	12.87±0.45
	MO	10.63±1.4	0.61±0.06	1.16±0.1	12.07±0.81
	PA	24.43±2.75	1.93±0.17	1.84±0.18	14.73±0.61
	LP	20.07±2.37	0.77±0.05	1.04±0.14	16.63±0.91
BOM	MS	16.93±1.44	0.78±0.15	1.69±0.16	19.64±0.48
	MO	11.50±1.7	0.78±0.18	1.51±0.29	15.23±0.25
	PA	27.93±3.95	2.89±0.26	2.96±0.37	17.10±0.57
	LP	22.87±2.45	0.99±0.14	1.51±0.15	18.55±0.64

Table S3. Plant physiological and biochemical indicators. Notes: *Lolium perenne* L. (LP), *Pennisetum alopecuroides* (L.) Spreng. (PA), *Melilotus officinalis* (L.) Lam. (MO), *and Medicago sativa* L. (MS). Control check (CK), biochar organic fertilizers (BO), biochar organic fertilizers and indigenous microorganisms (BOM). The values are expressed as the means (± standard error) from the experimental data (n = 3).

Processing group name	Landscape Grass Names	Chlorophyll content ($mg\cdot g^{-1}$)	PAL ($U\cdot g^{-1}$ FW)	POD ($U\cdot g^{-1}$ FW)	Soluble protein content ($mg\cdot g^{-1}$)
CK	MS	3.7±0.36	17.56±2.03	23.98±1.05	9.74±0.74
	MO	2.21±0.29	13.01±1.51	17.12±1.84	5.41±0.56
	PA	0.58±0.20	6.99±1.44	5.30±1.39	1.24±0.27
	LP	0.79±0.23	8.27±1.49	9.26±1.22	1.27±0.23
BO	MS	4.33±0.51	22.18±2.32	32.82±2.07	20.67±3.08
	MO	2.3±0.47	16.26±2.02	25.85±1.54	15.92±2.17
	PA	0.58±0.17	7.14±1.52	13.97±1.13	3.14±0.35
	LP	0.84±0.23	9.48±1.56	20.52±1.15	2.55±0.42
BOM	MS	4.44±0.61	29.01±2.88	35.51±2.37	28.91±1.62
	MO	3.31±0.39	22.04±2.03	28.23±2.06	16±2.15
	PA	0.91±0.33	8.86±1.54	18.15±1.49	3.28±0.73
	LP	0.99±0.39	10.02±1.87	21.13±1.78	3.48±0.67

Table S4. Nutrient content in plants. Notes: *Lolium perenne* L. (LP), *Pennisetum alopecuroides* (L.) Spreng. (PA), *Melilotus officinalis* (L.) Lam. (MO), *and Medicago sativa* L. (MS). Control check (CK), biochar organic fertilizers (BO), biochar organic fertilizers and indigenous microorganisms (BOM). The values are expressed as the means (± standard error) from the experimental data (n = 3).

Processing group name	Landscape Grass Names	N ($mg \cdot g^{-1}$)	P ($mg \cdot g^{-1}$)	K ($mg \cdot g^{-1}$)
CK	MS	5.88±1.12	1.27±0.25	7.43±1.64
	MO	5.7±1.07	1.4±0.22	8.59±2.25
	PA	21.18±2.53	2.80±0.32	47.53±7.62
	LP	22.39±3.15	2.61±0.31	49.09±9.28
BO	MS	6.42±1.49	1.35±0.23	9.6±2.49
	MO	6.45±2.17	1.46±0.31	10.54±2.76
	PA	25.58±3.23	3.03±0.43	54.14±8.14
	LP	27.51±2.56	2.87±0.58	53.19±8.25
BOM	MS	6.91±0.57	1.43±0.33	11.07±3.66
	MO	7.13±1.17	1.54±0.47	12.36±3.31
	PA	30.47±3.29	3.33±0.29	58.57±6.85
	LP	32.12±5.23	3.23±0.34	59.25±10.33

Table S5. Shannon-Wiener and Simpson Diversity Index. Notes: *Lolium perenne* L. (LP)*, Pennisetum alopecuroides* (L.) Spreng. (PA)*, Melilotus officinalis* (L.) Lam. (MO)*, and Medicago sativa* L. (MS). Control check (CK), biochar organic fertilizers (BO), biochar organic fertilizers and indigenous microorganisms (BOM). The values are expressed as the means (± standard error) from the experimental data (n = 4).

Processing group name	Time（day）	Landscape Grass Names	Shannon-Wiener Diversity index	Simpson Diversity Index.
CK	0		2.55±0.07	0.9923±0.0002
	10	PA	2.61±0.05	0.9884±0.0008
		LP	2.8±0.03	0.9854±0.0004
		MO	2.71±0.06	0.9898±0.0006
		MS	0.72±0.08	0.9865±0.0006
	20	PA	3.23±0.04	0.9872±0.0010
		LP	3.25±0.05	00.9839±0.0006
		MO	3.28±0.08	0.9851±0.0012
		MS	3.14±0.04	0.9850±0.0010
	30	PA	3.34±0.07	0.9841±0.0014
		LP	3.27±0.05	0.9804±0.0007
		MO	3.33±0.04	0.9871±0.0008
		MS	3.44±0.03	0.9826±0.0008
BO	0		2.83±0.02	0.9909±0.0001
	10	PA	3.07±0.04	0.9859±0.0005
		LP	3.10±0.06	0.9845±0.0004
		MO	2.90±0.0.9	0.9831±0.0004
		MS	3.04±0.08	0.9820±0.0004

	20	PA	3.40±0.04	0.9834±0.0008
		LP	3.42±0.06	0.9821±0.0014
		MO	3.23±0.03	0.9817±0.0012
		MS	3.51±0.02	0.9838±0.0010
	30	PA	3.37±0.05	0.9832±0.0010
		LP	3.46±0.02	0.9812±0.0015
		MO	3.55±0.02	0.9781±0.0009
		MS	3.49±0.05	0.9794±0.0007
BOM	0		2.92±0.07	0.9922±0.0001
	10	PA	3.11±0.09	0.9884±0.0008
		LP	3.11±0.04	0.9883±0.0006
		MO	3.03±0.06	0.9831±0.0004
		MS	3.15±0.03	0.9871±0.0007
	20	PA	3.41±0.08	0.9870±0.0015
		LP	3.43±0.08	0.9881±0.0008
		MO	3.34±0.08	0.9872±0.0007
		MS	3.51±0.09	0.9848±0.0004
	30	PA	3.45±0.07	0.9830±0.0006
		LP	3.46±0.07	0.9872±0.0003
		MO	3.54±0.05	0.9869±0.0009
		MS	3.56±0.1	0.9890±0.0005
